# Herbal Remedies and Their Possible Effect on the GABAergic System and Sleep

**DOI:** 10.3390/nu13020530

**Published:** 2021-02-06

**Authors:** Oliviero Bruni, Luigi Ferini-Strambi, Elena Giacomoni, Paolo Pellegrino

**Affiliations:** 1 Department of Developmental and Social Psychology, Sapienza University, 00185 Rome, Italy; 2 Department of Neurology, Ospedale San Raffaele Turro, 20127 Milan, Italy; ferinistrambi.luigi@hsr.it; 3 Sleep Disorders Center, Vita-Salute San Raffaele University, 20132 Milan, Italy; 4 Department of Medical Affairs, Sanofi Consumer HealthCare, 20158 Milan, Italy; Elena.Giacomoni@sanofi.com (E.G.); Paolo.Pellegrino@sanofi.com (P.P.)

**Keywords:** gamma-aminobutyric acid, GABA receptors, sleep, insomnia, herbal medicine

## Abstract

Sleep is an essential component of physical and emotional well-being, and lack, or disruption, of sleep due to insomnia is a highly prevalent problem. The interest in complementary and alternative medicines for treating or preventing insomnia has increased recently. Centuries-old herbal treatments, popular for their safety and effectiveness, include valerian, passionflower, lemon balm, lavender, and Californian poppy. These herbal medicines have been shown to reduce sleep latency and increase subjective and objective measures of sleep quality. Research into their molecular components revealed that their sedative and sleep-promoting properties rely on interactions with various neurotransmitter systems in the brain. Gamma-aminobutyric acid (GABA) is an inhibitory neurotransmitter that plays a major role in controlling different vigilance states. GABA receptors are the targets of many pharmacological treatments for insomnia, such as benzodiazepines. Here, we perform a systematic analysis of studies assessing the mechanisms of action of various herbal medicines on different subtypes of GABA receptors in the context of sleep control. Currently available evidence suggests that herbal extracts may exert some of their hypnotic and anxiolytic activity through interacting with GABA receptors and modulating GABAergic signaling in the brain, but their mechanism of action in the treatment of insomnia is not completely understood.

## 1. Introduction

Sleep is a fundamental physiological process required to maintain physical and emotional well-being. Healthy sleep is a crucial process for optimal cognitive performance, including attention, emotional reactivity, and learning and memory [1]. Sleep also contributes to a wide range of other physiological processes, e.g., metabolic and endocrine health and the strengthening of the immune system [2,3]. Chronic insomnia affects people across all geographies, socioeconomic levels, and cultures; because of this, sleeping pills are among the most frequently prescribed medicines worldwide [1,2].

It should be noted that multiple approved therapies for insomnia come with a safety warning, and some hypnotics (including, for example, barbiturates) have been abandoned because of unfavorable adverse event profiles or substance abuse [4]. In contrast, most herbal medicines for insomnia and anxiety offer an exceptional safety profile, sometimes with tenfold fewer adverse events than with pharmacotherapy [5,6,7]. Recent surveys suggest that almost two-thirds of individuals with sleep problems do not consult their doctor but search for treatment advice online, and herbal medicine remains a popular choice [8,9,10,11]. A number of recent studies demonstrate a steady increase in the uptake of complementary and alternative medicines for insomnia; the reasons for this increase may include dissatisfaction or concern for side effects with pharmacological treatment, previous positive experiences, and self-perceived effectiveness of alternative medicine [9,11,12].

Although their effectiveness is heavily debated, several herbal therapies for insomnia have been used for centuries, and many products, including valerian (*Valeriana officinalis* L.) and chamomile (*Matricaria* sp.), are still widely used today because of their good safety profile and their proposed anxiolytic and sedative proprieties [10,13,14,15,16,17].

Pharmacologically, herbal and traditional medicines represent complex mixtures of hundreds of constituents, making it difficult to isolate the active components and determine their exact mechanism of action [18]. Studies of several herbal remedies used for insomnia highlighted that changes in the central GABAergic neurotransmission could be responsible for the anxiolytic and the sedative properties of these remedies [13]. This is not surprising, as gamma-aminobutyric acid (GABA) is recognized as one of the main neurotransmitters responsible for sleep regulation. GABA_A_ receptor modulation is one of the four key mechanisms of action of the approved pharmacological therapies for insomnia (the other three mechanisms are melatonin receptor agonism, histamine 1 receptor antagonism, and hypocretin/orexin antagonism) [1].

The aim of this review is to summarize the current knowledge of the GABA receptors in sleep regulation and to perform a systematic analysis of literature addressing the GABAergic mechanisms of action of herbal remedies for insomnia.

## 2. Stages of Sleep

Three distinct vigilance states can be identified on the basis of the level of arousal and electroencephalogram (EEG) activity: wakefulness, non-rapid eye movement (NREM), and rapid eye movement (REM) [2]. Healthy, young individuals usually experience several NREM and REM cycles during the night; the typical length of one NREM–REM cycle in humans is approximately 90 min [2,19].

The three vigilance states are regulated by wakefulness-promoting, NREM-promoting, and REM-promoting distinct neuronal groups (nuclei) located in the basal forebrain, thalamus, and brainstem [2,20]. Brain nuclei promoting different vigilance states exert reciprocally inhibitory activity and are involved in modulating the activity of numerous other structures of the central nervous system [2,20,21].

Wakefulness is regulated by a number of different neurotransmitter systems, including acetylcholine, serotonin, norepinephrine, histamine, orexins, neuropeptide S, dopamine, glutamate, and even GABA [2]. In the brainstem, the pontine locus coeruleus promotes wakefulness via excitatory connections to the cerebral cortex and inhibitory connections to sleep-promoting nuclei [22]. The alternation of NREM and REM sleep phases during the night is likely controlled by several mechanisms, including a reciprocal interaction of “REM-on” glutamatergic neurons in the pontine/mesencephalic reticular formation and “REM-off” norepinephrine/serotonin neurons in the dorsal raphe and the locus coeruleus [2,20]. Transition between vigilance states is orchestrated by the central pacemaker: the suprachiasmatic nucleus [20,23]. As the most widespread inhibitory neurotransmitter in the brain, GABA plays a role in inhibiting both “REM-on” and “REM-off” neurons in the brainstem and in regulating transitions between REM sleep and wakefulness or NREM sleep [2,23]. In addition, various groups of GABA neurons outside the brainstem are involved in the control of circadian timing and homeostatic regulation of sleep [2].

## 3. The Role of GABAergic Signaling in Sleep Physiology

As a major inhibitory neurotransmitter, GABA helps maintain the overall balance of neuronal excitation and inhibition in the central nervous system and plays one of the central roles in brain development and function [24]. Over 20% of all neurons in the brain are estimated to be GABAergic [25]. Three different GABA receptors, GABA_A_, GABA_B_, and GABA_C_, are involved in the regulation of sleep and arousal (albeit to different extents) [2,25]. The most commonly used hypnotics exert their effect on GABA systems, most notably through allosteric modulation of the benzodiazepine site [26,27,28]. Similarly, many herbal medicines have been proposed to enhance GABAergic signaling, many through interactions with the GABA_A_ receptor [13].

### 3.1. GABA_A_ Receptor

**Figure 1:**
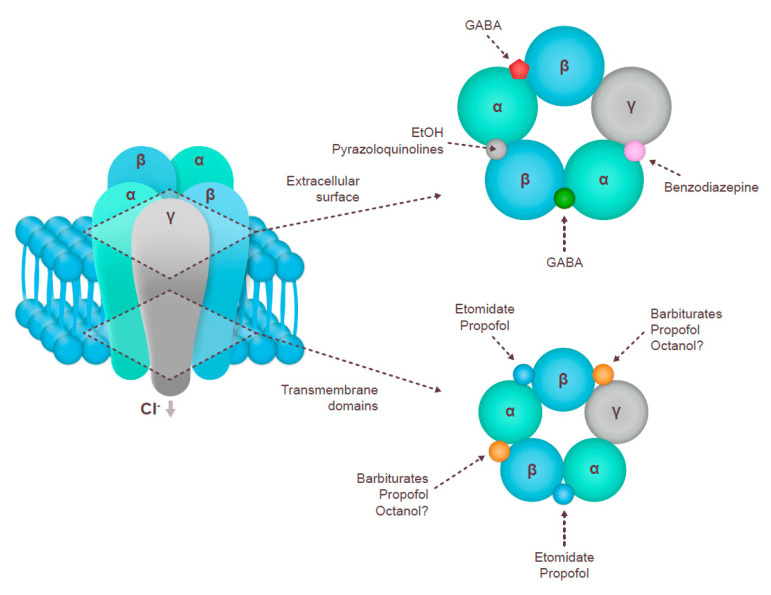
The structure of the GABA_A_ receptor and the location of common agonist and antagonist binding sites [26,29,30,31]. Cl^−^, chlorine ions; GABA, gamma-aminobutyric acid; EtOH, ethanol.

The fast-acting ionotropic GABA_A_ receptors were the first to be discovered and have been the target of three generations of anxiolytics and hypnotics [1,2,29]. GABA_A_ receptors are pentameric, ligand-gated Cl^–^ ion channels; the classical synaptic subtypes are formed of two α, two β, and one γ or δ subunit, the α1β2γ2 receptor being the most abundant [29,30] (Figure 1).

Barbiturates were the first generation of sedative/hypnotic drugs introduced in the early 20th century. Their binding site on the GABA_A_ receptor is different from that of GABA, and they act via direct activation of the receptor. Barbiturates do not show selective affinity to different receptor compositions of GABA_A_. Second-generation sleep aids (benzodiazepines) are GABA_A_ allosteric modulators that bind to the interface between the α and the γ subunits across a range of receptor compositions [27,29,31]. Recently developed third-generation non-benzodiazepine hypnotics include, among others, zopiclone (a cyclopyrrolone), zolpidem (an imidazopyridine), and zaleplon (a pyrazolopyrimidine), which are sometimes collectively called the “Z drugs” [32]. All GABA_A_ agonists help with entering and maintaining sleep by suppressing REM sleep and lower frequency waves while promoting high frequency waves [19]. The effects of GABA_A_ agonists on sleep stages may vary; for example, eszopiclone does not have any effect on the length of NREM or REM sleep [33]. Reductions in theta and alpha frequencies have been observed in older, but not in young, adults with zolpidem; moreover, zolpidem decreased Stage 1 NREM in older adults, with no other age-related changes in sleep parameters [34].

### 3.2. GABA_B_ Receptor

**Figure 2:**
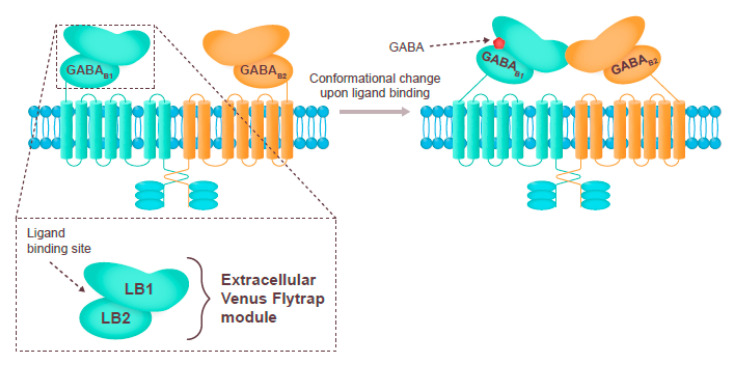
The structure of the GABA_B_ receptor, its ligand binding site, and the downstream signaling elements [39,40]. GABA, gamma-aminobutyric acid; LB, ligand binding.

GABA_B_ receptors are slow-acting metabotropic G-protein-linked dimers containing one GABA_B1_ (GABA_B1a_ or GABA_B1B_) and one GABA_B2_ subunit [25,28,29] (Figure 2). Fewer drugs have been developed to target the GABA_B_ receptor, baclofen being the most popular agonist, and there are less clinical data available than for the GABA_A_ receptor [27,35]. Although GABA_B_ agonists may promote sleep by increasing the duration of NREM and REM sleep, the effect is believed to be largely off-target [28,36]. Binding to the GABA_B_ receptor may be responsible for the sleep-promoting effects of the drug gamma hydroxybutyrate. Activation of GABA_B_ receptors on hypocretin/orexin neurons increases the power and duration of slow wave sleep and decreases the frequency of transitions between wakefulness and REM sleep [37,38].

### 3.3. GABA_C_ Receptor

**Figure 3:**
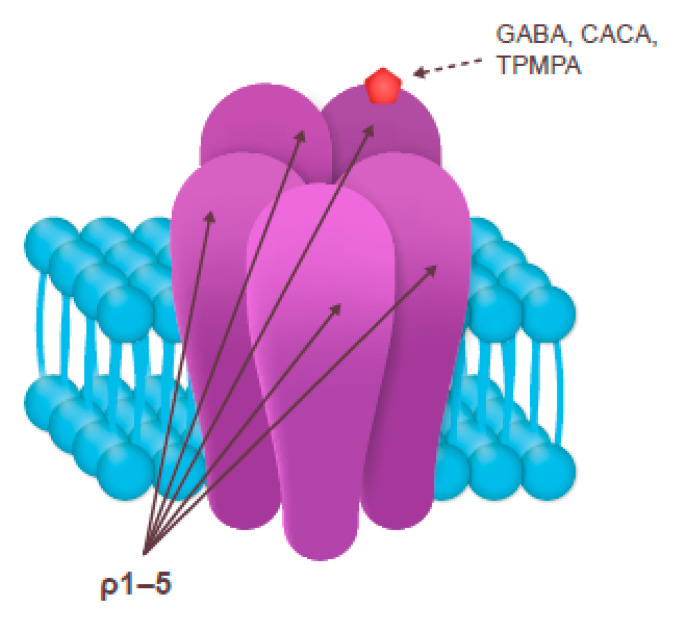
The structure and the ligand-binding site of the GABA_C_ receptor [41]. CACA, cis-4-aminocrotonic acid; GABA, gamma-aminobutyric acid; ρ1–5, GABA_C_ ρ subunits 1–5; TPMPA, (1,2,5,6-tetrahydropyridin-4-yl) methylphosphinic acid.

The subclass of GABA_A_ receptors containing ρ subunits is often called GABA_C_ or GABA-ρ; they belong to the same family of fast-acting pentameric, ligand-gated Cl^–^ ion channels as GABA_A_ [25,27,41] (Figure 3). Although both GABA_A_ and GABA_C_ receptors bind GABA, they have separate sets of agonists and antagonists. GABA_C_ receptors are more sensitive to GABA than the other two receptor subclasses [25].

A selective GABA_C_ antagonist (1,2,5,6-tetrahydropyridin-4-yl) methylphosphinic acid (TPMPA) has been shown to decrease the relative duration of NREM and REM sleep in rats [42]. In contrast, the selective partial GABA_C_ agonist cis-4-aminocrotonic acid (CACA) does not have any effect on the relative duration of REM sleep [43].

A number of studies suggest that different classes of GABA receptors may play varying roles in sleep control, e.g., promoting different phases of sleep [25]. The expression pattern of each class and the cellular localization (synaptic or extrasynaptic) may play a role in the extent of the receptor involvement in sleep control [19]. This involvement may be influenced by other physiological and pathological conditions; for example, sleep deficits in slow wave sleep recorded in patients with schizophrenia may be specifically linked to the GABA_B_ receptor [44]. Although most of the currently available hypnotics target GABA_A_, ongoing research on the physiology and pharmacology of the other two types of GABA receptors may lead to development of therapies for insomnia targeting GABA_B_ or GABA_C_.

## 4. Herbal Remedies Acting on GABA Metabolism and Function

Herbal medicine, i.e., applications of plants or plant-derived materials for therapeutic purposes, has been used for centuries to treat a range of sleep disorders; notable examples include valerian (*Valeriana officinalis* L.), passionflower (*Passiflora incarnata* L.), lemon balm (*Melissa officinalis* L.), and Californian poppy (*Eschscholzia californica Cham.*) [13,16]. More recently, therapies being tested for efficacy in insomnia have included combinations of herbal extracts with melatonin and vitamin B6 [45]. A growing body of recent research has been dedicated to dissecting the content of naturally derived sleep aids and to determining the specific compounds responsible for their sedative properties. Multiple mechanisms of actions have been proposed, including those that promote GABAergic signaling, most commonly through an interaction of the active component with the GABA_A_ receptor [6,13,46].

### 4.1. Systematic Literature Review

We have searched PubMed and Google Scholar for publications describing GABAergic effects of herbal medicines and their active components that could explain their mechanism of action in sleep regulation. The search terms included (“herbal medicine” OR “herb”) AND (“GABA” OR “gammaaminobutyric acid” OR “gamma aminobutyric acid”) AND (“sleep” OR “hypnotic” OR “sedative”).

**Figure 4:**
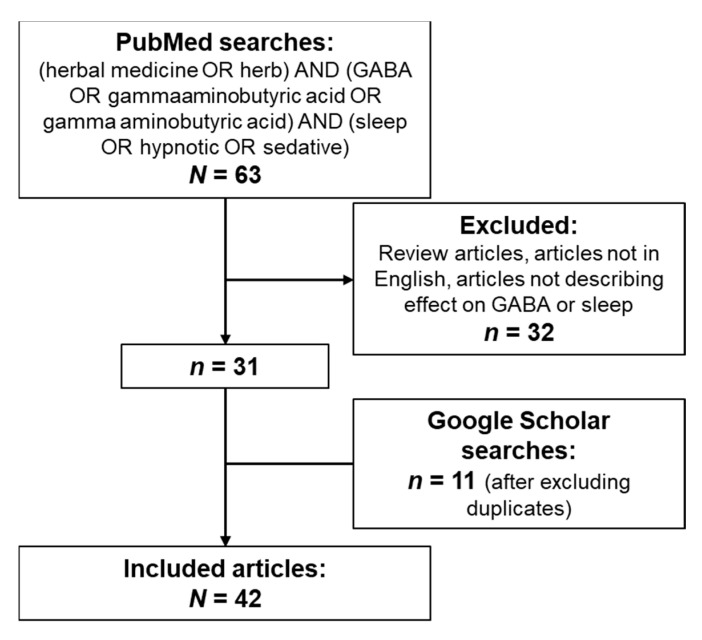
PRISMA flow diagram. GABA, gamma-aminobutyric acid; PRISMA, preferred reporting items for systematic reviews and meta-analyses.

The PubMed search returned 63 results; after removing reviews, articles not in English, and studies that did not assess GABAergic effects or sleep, 31 results were included. A number of additional publications were identified through Google Scholar searches; after removing duplicates, 11 additional articles were added to the reference library (Figure 4).

### 4.2. Natural Compounds Acting on GABA_A_, GABA_B_, and GABA_C_

We analyzed the articles identified in the literature search for the description of specific mechanisms of action targeting GABAergic signaling in sleep. The results of the literature analysis are shown in Table 1.

The vast majority of herbal medicines acted through the GABA_A_ receptor (mostly via binding to the GABA or benzodiazepine sites) (Figure 1). The specific chemicals that serve as natural modulators of the GABA_A_ receptor (alkanes and alkaloids, flavones, flavonoids and isoflavonoids, phenols, terpenes, coumarins, etc.) have been described in detail in a recent review that addressed the specific pharmacological features of their interactions with the receptor [46]. Here, we present a broader summary of herbal extracts that may be used to regulate sleep, possibly acting via GABAergic signaling.

The largest body of evidence for GABA_A_ modulation is associated with valerian root (*Valeriana officinalis* L.), which is widely used to reduce the latency of sleep onset and increase sleep quality [13,79]. Valerian root extract contains over 150 chemical constituents including alkaloids, terpenes, organic acids and their derivatives, valepotriates and flavones [13,48]. GABA itself may be present in the valerian extracts, although its bioavailability is questionable [5]. Notably, small differences have been reported between extracts from plants grown in different conditions or processed in a different manner, and large-scale producers have standardized protocols of plant growth and extract preparation aimed at reducing variability [48]. Studies in tissue culture and animal models suggest that components of valerian extract (*Valeriana officinalis* L.) possess prominent dose-dependent GABA_A_ agonistic activity [49,51]. 6-methylapigenin is a potent positive modulator of GABA_A_, possibly binding to the benzodiazepine site at the interface of α and γ subunits, whereas valerenic acid and valerenol have been shown to interact with the β subunit of the receptor [47,48].

*Magnolia* sp., *Artemisia* sp., Chinese magnolia vine (*Schisandra chinensis*), lotus (*Nelumbo nucifera*), and drumstick tree (*Moringa oleifera*) have all been shown to contain GABA_A_ agonists that promote sleep in various animal models. A potent GABAergic effect via the GABA_A_ receptor (benzodiazepine site) has been demonstrated for herbal mixes used in traditional medicine in Japan (yokukansan) and China (suanzaorentang); however, the specific herbs and compounds responsible for the effect remain to be identified [76,78]. A number of different approaches can be used to identify these compounds; for example, in one study, an in silico screen of a traditional Chinese medicine library was performed and found that 2-O-caffeoyl tartaric acid, 2-O-feruloyl tartaric acid, and mumefural are potent GABA_A_ receptor agonists at both GABA and benzodiazepine binding sites [80]. Tartaric acid derivatives are present in various fruit syrups and juices, and mumeferal is derived from the processed fruit of Japanese apricot (*Prunus mume Sieb. et Zucc.*) (a traditional health food) [81].

Extract of dried flowers of chamomile (*Matricaria* sp.) has been used as a mild tranquilizer and sleep inducer for thousands of years and contains 28 terpenoids and 36 flavonoids [82,83]. Among them, apigenin has been shown to exhibit a hypnotic activity by activating the GABA_A_ receptor at the benzodiazepine binding site [26,46,82]. Apigenin is an active component of several herbal sleep remedies such as passionflower (*Passiflora incarnata* L.), which is used to reduce sleep latency and increase sleep duration [13,46]. Other GABA_A_ allosteric modulators acting at the benzodiazepine site include alkaloids isolated from the California poppy (*Eschscholzia californica Cham.*), which is used to induce relaxation and sleep [69].

There is much less evidence of herbal medicines interacting with the GABA_B_ or the GABA_C_ receptors. The extract of *Passiflora incarnata* has been shown to inhibit the binding of ligands to both GABA_A_ and GABA_B_ receptors in a concentration-dependent manner, suggesting that it contains antagonists of both receptor subtypes [65]. Notably, *Passiflora incarnata* L. extract contains a high amount of GABA and therefore has a potential to exert its hypnotic activity through all three types of GABA receptors, although its exact mechanism of action remains to be demonstrated [66]. The aqueous root extract of Indian ginseng (*Withania somnifera* L.) has been shown to act as a potent agonist of the GABA_C_ receptor in addition to weakly activating GABA_A_ [67]. Various natural compounds have been implicated in the plant’s mechanism of action, including withanone, withaferin A, and triethylene glycol [67,84].

### 4.3. Other Mechanisms of Action Related to GABA Signaling

Several indirect effects on GABA signaling have been reported for various medicinal plant extracts. Valerian root extract (*Valeriana officinalis* L.) may mediate inhibition of enzymatic destruction of GABA, increasing GABA availability [5]. Extract of *Melissa officinalis* L. decreases the level of GABA transaminase in hippocampal neurons [72]. Unidentified components of a Mexican tree *Ternstroemia* *lineata* DC. have been shown to promote GABA release in mouse brain slices [85]. Tenufolin, the active component of *Polygala* *tenuifolia*, increases the expression of GABA transporter 1 and GABA availability in animal models [70,71]. Activation of GABA synthesis through enhanced expression of glutamic acid decarboxylase (GAD) has been demonstrated for sanjoinine A, an alkaloid isolated from jujube (*Zizyphus* *jujuba*) [63]. Finally, although the *Citrus* *aurantium* essential oil exerts its anxiolytic effect via the serotonin receptor, an indirect effect on GABAergic system has been described as well [75,86]. These results suggest that herbal sleep medicines may have a plethora of direct and indirect effects on GABAergic signaling beyond direct interaction with GABA receptors.

## 5. Discussion and Conclusions

Insomnia is a widespread, often chronic, disorder that affects 5–15% of the general population and is associated with a great reduction in quality of life [1,2,87]. Among prescription medicines for insomnia, many therapies act via modulation of GABAergic signaling, including potent hypnotics such as benzodiazepines and “Z drugs” that bind to various sites on the GABA_A_ receptor [1,27,30]. Although GABA_B_ and GABA_C_ receptors have distinct roles in controlling various stages of sleep, none of the currently approved prescription medicines target these receptor subtypes; however, ongoing research may lead to the development of such medicines in the future.

The ability of herbal extracts to reduce sleep latency, increase sleep duration, and improve sleep quality has been explored in numerous studies; however, robust clinical evidence supporting their use for the treatment of insomnia is currently lacking, emphasizing the need for research in this area [87,88]. Mechanistic studies have shown that herbal medicines used for the treatment of depression, anxiety, and insomnia may exert their effect through various mechanisms of action. Components of ginseng (*Withania somnifera* L.), *Ginkgo biloba* L., and St John’s Wort (*Hypericum perforatum* L.) have been shown to influence the reuptake of neurotransmitters, such as norepinephrine, dopamine, and serotonin [70,89,90]. Extracts of jujube seeds and valerian (*Valeriana officinalis* L.) directly interact with serotonin receptors [64,89], and *Griffonia simplicifolia Baill.* contains 5-hydroxytryptophan, a natural precursor of serotonin [91]. L-theanine, which is found in green tea, has been discovered to potentiate GABA, dopamine, and serotonin receptors and to inhibit glutamate reuptake [92]. Active components of lavender (*Lavandula angustifolia Miller*) can bind the glutamate N-methyl-D-aspartate receptors and serotonin transporters [93]. Finally, several herbal substances may interact with glutamic acid decarboxylase or modulate GABA and serotonin receptors [6,88,94]. Sleep-promoting GABAergic neurons represent the main cellular target of pharmacological therapies for insomnia, and GABA signaling appears to be the target of a large number of over-the-counter herbal sleep aids [1,2,13]. The exceptional safety profile of herbal medicines, especially when compared with pharmacotherapy for insomnia, and their wide acceptance by patients, serve as a strong argument in favor of further investigations that aim to define their mechanism of action more precisely and that aim to confirm their clinical efficacy in terms of specific sleep parameters.

In conclusion, despite the availability of multiple hypnotic drugs, side effects remain an issue, and there is ongoing demand for safer treatment options for insomnia. The evidence reviewed here suggests that multiple plant-derived substances may serve as sleep aids by modulating GABAergic signaling in the brain. The exceptional safety profile of herbal medicines and their wide acceptance by patients serve as a strong argument in favor of further investigations of their mechanism of action and identification of specific compounds that exert the hypnotic effect.

## Figures and Tables

**Table 1:** Common medicinal plants with known sleep-inducing properties that target GABAergic signaling.

Latin and Common Name	Known Chemical Components	Known Effect on Sleep	Target	Model	References
	**Individual Plants**				
*Valeriana officinalis* L. (Valerian)	Alkaloids, terpenes, organic acids and their derivatives, valepotriates, and flavones	Reduces sleep latency, improves subjective measures	GABA_A_ receptor	In vitro studies; clinical studies	[47,48,49,50,51]
*Magnolia* sp.	Magnolol and honokiol	Promotes REM sleep	GABA_A_ receptor	In vitro studies; i.p. administration in mice	[52,53,54]
*Schisandra chinensis* *(Turcz.) Baill.* (Chinese magnolia-vine, Magnolia berry)	Schizandrin B	Promotes sleep	GABA_A_ receptor	i.p. administration in mice and male rats	[55,56,57]
*Artemisia* sp.	Benzodiazepines	Reduces sleep latency	GABA_A_ receptor	In vitro studies; i.p. administration in male mice	[58,59]
*Nelumbo nucifera* *Gaertn.* (Lotus)	Nuciferine, alkaloids	Promotes sleep	GABA_A_ receptor	In vitro studies	[60]
*Moringa oleifera* *Lam.* (Drumstick tree)	Oleic acid, β-Sitosterol, and Stigmasterol	Increases sleep quality	GABA_A_ receptor	p.o. administration in male mice	[61]
*Piper methysticum* L. (Kava-kava)	Kavapyrones	Decreases sleep latency; no effect on NREM sleep	GABA_A_ receptor (not benzodiazepine site)	p.o. administration in mice	[62]
*Zizyphus jujube* (Jujube, or red date)	Sanjoinine A, suanzaorentang	Improves sleep quality, prolonging sleep time and increasing NREM sleep	GABA_A_ receptor; activation of GABA synthesis through enhanced expression of GAD; serotonin receptors	i.p. and p.o. administration in male rats	[63,64]
*Passiflora incarnata* (Passionflower)	Apigenin, alkaloids, flavones	Reduces sleep latency, increases sleep duration	GABA_A_ and GABA_B_ receptors, (and possibly GABA_C_ receptor)	In vitro studies; p.o. administration in mice	[65,66]
*Withania somnifera* L. (Indian ginseng)	Withanolide A, withaferin A	Reduces sleep latency, improves sleep quality	GABA_A_ and GABA_C_ receptors	In vitro studies; clinical studies	[67,68]
*Eschscholzia californica Cham.* (Californian poppy)	Alkaloids	Improves sleep latency and duration	GABA_A_ receptor; serotonin receptor	In vitro studies	[69]
*Polygala tenuifolia* *Willd.* (Yuan Zhi)	Tenufolin	Increases sleep duration	Increases the levels of GABA and GABA transporter 1	Zebrafish and rats	[70,71]
*Melissa officinalis* L. (Lemon balm)	Rosmarinic acid	Improves sleep quality	Decreases the level of GABA transaminase	In vitro studies; i.p. administration in mice	[72]
*Ginkgo biloba* L. (Ginkgo)	Ginkgotoxin, flavonoids, terpenoids	Improves subjective sleep quality measures	Inhibition of GAD activity	Clinical studies	[73]
*Hypericum perforatum* L. (St John’s Wort)	Hypericin, pseudohypericin, hyperoside, among others	Increases REM latency and deep sleep	Inhibition of GAD and GABA transporter activity	Clinical studies	[74]
*Citrus aurantium* L. (bitter orange)	Limonene, β-myrcene	Increases sleep duration	Serotonergic system; proposed interaction with GABA receptor binders, such as diazepam	p.o administration in male mice	[75]
**Plant mixes**					
Yokukansan (Atractylodes lancea rhizoma, Poria sclerotium, Cnidium rhizoma, Japanese Angelica radix, Bupleurum radix, Glycyrrhiza radix, and Uncaria thorn)	Various	Decreases sleep latency, improves dream content in the REM behavior disorder	GABA_A_ receptor	p.o. administration in male mice; clinical studies	[76,77]
Suanzaorentang, a traditional Chinese medicine	Various	Increases NREM, no effect on REM sleep	GABA_A_ receptor; serotonergic system	Clinical studies	[64,78]

Herbal medicines were selected if their proposed mechanism of action involved GABA synthesis, transport, or receptors. GAD, glutamic acid decarboxylase; GABA, gamma-aminobutyric acid; i.p., intraperitoneal; NREM, non-rapid eye movement; p.o., oral; REM, rapid eye movement.

## Data Availability

The study did not report any data; all references used in the review are included in the reference list.
